# Development of Toolkit for Non-Pharmacological Pain Management in the Austrian Living Lab

**DOI:** 10.1093/geroni/igaf122.327

**Published:** 2025-12-31

**Authors:** Daniela Schoberer, Manuela Hoedl, Eva Pock, Doris Eglseer

**Affiliations:** Medical University of Graz, Graz, Steiermark, Austria; Medical University of Graz, Graz, Steiermark, Austria; Medical University of Graz, Graz, Steiermark, Austria; Medical University of Graz, Graz, Steiermark, Austria

## Abstract

The OPINION Lab, established as a Living Lab in an Austrian nursing home, aimed to develop a practical, evidence-based toolkit for non-pharmacological pain management, addressing the high prevalence of chronic pain, especially among residents with dementia. In this Living Lab, researchers, caregivers, residents, and family members collaboratively engaged in the process of developing and testing the toolkit, ensuring that the interventions were relevant and feasible for everyday nursing practice. The study followed a systematic, participatory approach, incorporating nursing staff from the beginning to identify relevant interventions, synthesize current evidence, and assess the acceptability, feasibility, and required resources for implementation. Rapid reviews were conducted for interventions lacking sufficient evidence, using Cochrane Rapid Reviews guidelines. The toolkit includes 20 non-pharmacological interventions, addressing key aspects such as effectiveness, indications, application areas, and required resources. This method of participatory research within the OPINION Lab encouraged close collaboration between nursing researchers and caregivers, facilitating a strong connection between theory and practice. The toolkit was developed to be user-friendly, ensuring its practical applicability in the nursing home setting. It is currently being implemented and evaluated under scientific supervision. The participatory approach at the OPINION Lab has not only resulted in an evidence-based toolkit but also enhanced mutual understanding between research and practice, supporting a holistic, patient-centered approach to pain management in nursing homes.

